# van der Waals dispersion interactions in molecular materials: beyond pairwise additivity

**DOI:** 10.1039/c5sc00410a

**Published:** 2015-03-30

**Authors:** Anthony M. Reilly, Alexandre Tkatchenko

**Affiliations:** a The Cambridge Crystallographic Data Centre , 12 Union Road , Cambridge , CB2 1EZ , UK; b Fritz-Haber-Institut der Max-Planck-Gesellschaft , Faradayweg 4-6 , Berlin 14195 , Germany . Email: tkatchen@fhi-berlin.mpg.de ; Tel: +49 3084134802

## Abstract

van der Waals (vdW) dispersion interactions are a key ingredient in the structure, stability, and response properties of many molecular materials and essential for us to be able to understand and design novel intricate molecular systems. Pairwise-additive models of vdW interactions are ubiquitous, but neglect their true quantum-mechanical many-body nature. In this perspective we focus on recent developments and applications of methods that can capture collective and many-body effects in vdW interactions. Highlighting a number of recent studies in this area, we demonstrate both the need for and usefulness of explicit many-body treatments for obtaining qualitative and quantitative accuracy for modelling molecular materials, with applications presented for small-molecule dimers, supramolecular host–guest complexes, and finally stability and polymorphism in molecular crystals.

## Abstract 2

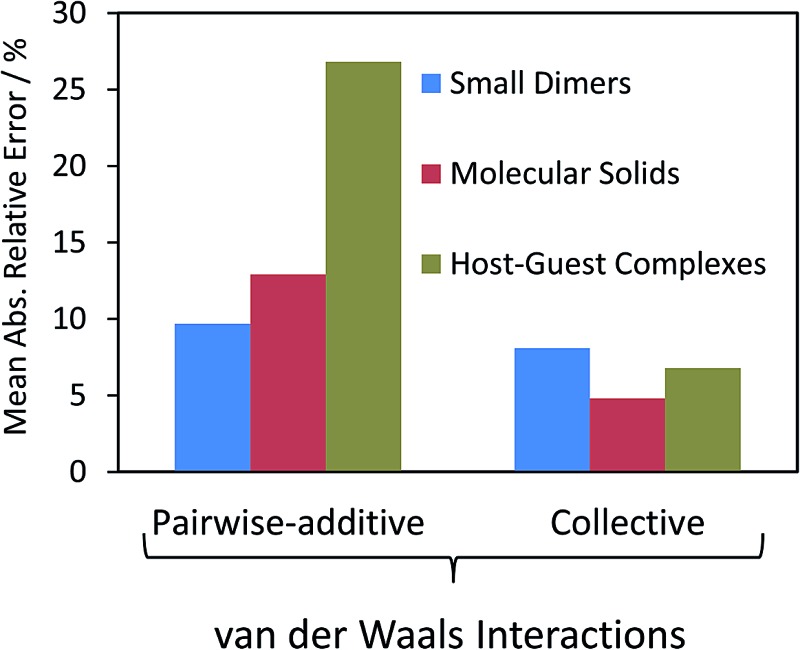
In this perspective we discuss recent advances in the understanding of collective and many-body van der Waals interactions and their role and impact for molecular materials.

## 1 Introduction

The study of intermolecular or non-covalent interactions has long been key to our understanding of complex molecular systems, particularly when considering the assembly of molecules into supramolecular systems or condensed matter. Predicting and studying cohesion in molecular materials relies heavily on accurate and physically realistic treatments for the different intermolecular forces that arise in such materials, with those that result from instantaneous fluctuations of electrons, which are referred to as dispersion or van der Waals (vdW) interactions,†
†In the chemistry community van der Waals interactions may refer to any non-covalent interaction, whereas in the (chemical-) physics community the terms dispersion and van der Waals interactions are largely synonymous and are used interchangeably, as we will do here. being particularly important. Although they are typically weaker than electrostatic interactions or hydrogen bonding, their long-ranged nature and ubiquity in all molecular systems means they can always play an important role in non-covalent binding.^1,2^ Indeed, as they scale with system size, in some cases strongly non-linearly,^3^ their importance can grow for larger molecules and molecular assemblies.

Dispersion interactions are quantum-mechanical in nature and form part of the correlation energy of a system. They stem from the inherent zero-point fluctuations of electrons on an atom, which give rise to instantaneous multipole moments. These moments induce multipole moments on other atoms, which then interact with the original moment. The leading order contribution is from dipole–dipole interactions, with a 1/*R* ^6^ dependence on distance.

The ability of an atom to form such moments (both instantaneous and induced) depends on its polarisability. The polarisability of an atom is influenced by its chemical bonding but also by its environment through polarisation and induction effects, where it is changed by coupling to the electric field of its surrounding atoms. Such effects are known to be non-additive in nature,^1^ and are important for going beyond simple point charges in the modelling of electrostatics in force fields,^4,5^ and modelling molecular polarisabilities.^6^

However, despite the central role of polarisability in vdW interactions, the vast majority of models ignore its non-additive and non-local nature, using simple pairwise-additive models with dispersion coefficients that do not capture any dependence on non-local environment. In neglecting many-body effects, these methods often require empirical fitting of parameters to a given molecule and environment.^2,7^

Pairwise-additive descriptions of vdW interactions have proven qualitatively successful in numerous applications ranging from bio-molecular simulations to modelling crystallisation. However, in recent years there has been a surge in interest in developing more sophisticated and quantitatively accurate approaches. The challenge of structure prediction, including polymorphism in molecular crystals,^8,9^ and the availability of good-quality benchmark datasets^10–12^ have driven developments for more realistic and less empirical approaches to vdW interactions.

Many of the developments in our understanding of vdW interactions in molecular systems have taken place in the context of vdW-inclusive density-functional theory (DFT). Workhorse semi-local and hybrid density functionals neglect long-range correlation^13^ and considerable progress has been made in augmenting semi-local density functionals with non-local vdW contributions.^14–16^ However, many of these vdW-inclusive DFT approaches are still founded on pairwise models of dispersion interactions, as use of fully *ab initio* approaches, such the random-phase approximation,^15^ is not feasible due to their far higher computational cost than standard semi-local DFT.

Despite their successful application in many areas, including occasional successes for crystal-structure prediction,^17^ quantitative and qualitative failures remain when employing pairwise models of vdW interactions.^18–21^ As a result, in recent years there has been considerable interest in studying and modelling non-additive and many-body contributions to vdW interactions, which can take a number of forms.^22^ In this perspective article we highlight collective effects in vdW interactions in molecular materials, focusing on non-additivity in polarisabilities and methods that can capture these effects, in particular, the recently developed many-body dispersion (MBD) method.^23,24^ Using a number of applications, we illustrate new insights into the role of collective vdW effects in the stability and properties of molecule materials. For a more in-depth discussion of the theory and physics of many-body vdW interactions the reader is referred to recent papers concerning a number of different approaches to the problem.^15,24–27^

In the following section (Section 2) we briefly overview the theory of widely used pairwise descriptions of vdW interactions, before discussing the role of polarisation effects in vdW interactions and introducing a number of approaches to many-body van der Waals in Section 3. We illustrate the application of these methods to different systems in Section 4 before finally discussing the prospects and challenges for understanding and modelling collective vdW interactions in Section 5.

## 2 Pairwise-additive descriptions of van der Waals interactions

One of the most widespread pictures and models of vdW interactions is the pairwise-additive one, whereby the vdW energy of a collection of atoms or molecules can be expressed as summation of attractive contributions from localised multipoles:1
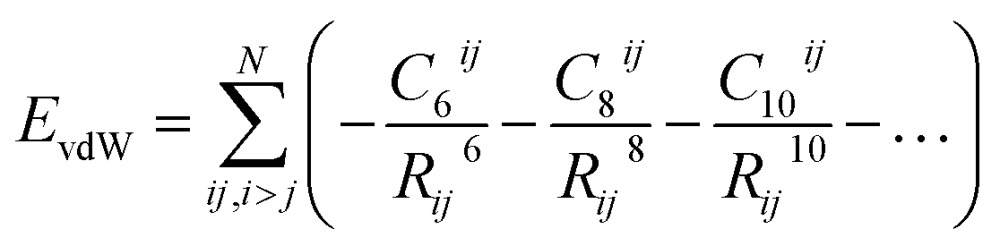
where *R*_*ij*_ is the interatomic or fragment separation and *C*_6_^*ij*^ is dipole–dipole dispersion coefficient for atoms or fragments *i* and *j*, with *C*_8_ representing dipole–quadrupole and *C*_10_ representing quadrupole–quadrupole and dipole–octupole dispersion coefficients. The dispersion coefficients are determined by the corresponding polarisabilities of the atoms or fragments, relating the extent to which valence electrons can respond to local electric-field fluctuations. For the leading-order *C*_6_ term the dispersion coefficient can be expressed as^1,28^
2
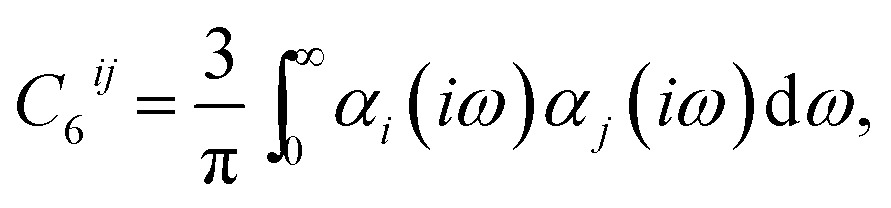
where *α*(*iω*) is the frequency-dependent dipole polarisability. Eqn (1) has its origin in second-order perturbation theory, where the vdW interactions of two *well-separated* atoms can be shown to have a 1/*R* ^6^ dependence. At small interatomic separations eqn (1) is clearly not physical, as the vdW energy diverges to –∞. This arises from its derivation for well-separated fragments at long ranges. As a practical solution, in nearly all applications of the pairwise model damping is required at short distances. The foundation of many empirical force fields is the Lennard-Jones potential,^7^ 
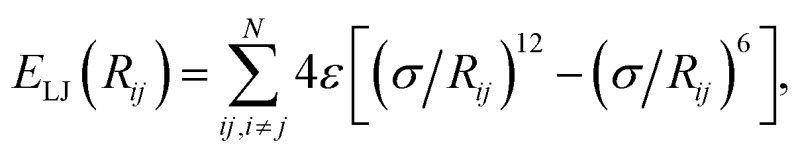
 where the parameter *σ* controls the balance between the attractive vdW term and a short-range repulsion term, which models effects such as Pauli repulsion, or direct overlap of electron clouds. In vdW-inclusive DFT, damping functions are commonly used to switch off the vdW interaction at short distances,^14,16^ not only due to this divergence but also because widely employed density functionals include short-range correlation, of which the vdW energy forms a part. These damping functions often contain one or two functional-specific parameters, which may control the distance at which the damping takes effect,^14,16^ or even scale some or all of the coefficients to “match” better with DFT energies.^29,30^

Within the pairwise description of van der Waals interactions the accuracy obtainable (*i.e.* ignoring potential non-additive or high-order contributions) is governed by the dispersion coefficients used, whether to include higher than dipole contributions, and the damping or short-range attenuation of the interaction. Empirical potentials typically employ sets of parameters fitted to broad classes of molecules, *e.g.* bio-molecules,^31^ organic molecules in a specific phase,^32,33^ or small groups of molecules.^34^ The fitting of such potentials can be highly empirical, with different terms in the potential compensating for deficiencies in other terms. Indeed, transferability of empirical potentials is a serious issue for this reason.^2^

In vdW-inclusive DFT, early approaches employed empirical parameter sets as well,^29,35^ but more recent approaches aimed at more physically grounded schemes for obtaining dispersion coefficients, depending on hybridisation of atoms or the local electron density.^30,36,37^ This is the first type of collective effect in vdW interactions,^22^ where crowding of an atom by its neighbours results in reduction of polarisabilities from ideal free-atom values. The Tkatchenko–Scheffler (TS) model (also referred to as the DFT + vdW approach) uses the local Hirshfeld volume of an atom (*V*_eff_) to reweigh accurate free-atom polarisabilities, *C*_6_ coefficients, and van der Waals radii for this effect:3
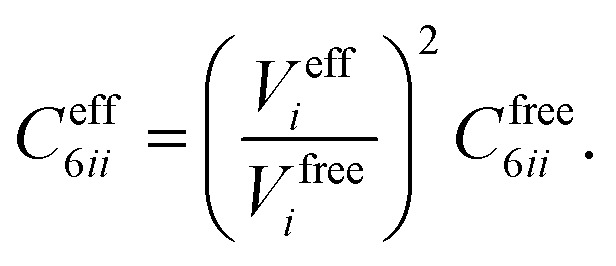

4
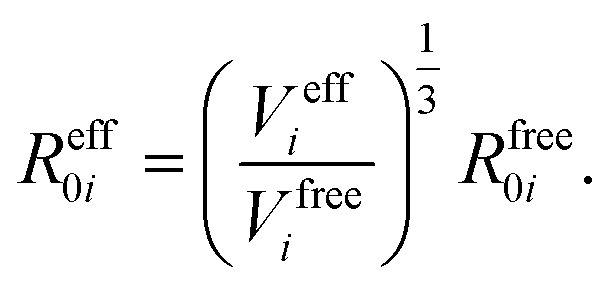

5
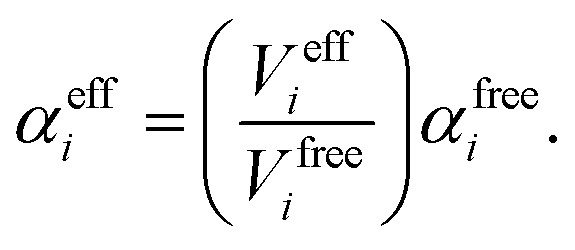



The resulting *C*_6_ coefficients are functionals of the local electron density and capture hybridisation effects well, with an accuracy of 5.5% for small molecules.^36^ When used in DFT a damping function is required, with a single parameter fitted to the functional employed. Given its small-molecule accuracy and its ability to capture local effects on vdW interactions, we will use TS as a benchmark to illustrate the importance of many-body and collective effects. Other approaches, such as atom-centred potentials^38^ and self-consistent vdW density functionals^39^ are also employed in DFT: the interested reader is referred to a number of reviews on vdW-inclusive DFT.^14,16,40^

For sufficiently small or weakly polarisable systems, the pairwise-additive approximation is of enormous use and can be remarkably accurate. However, most molecular systems of interest are neither small nor weakly polarisable. Furthermore, in many instances it is *differences* in vdW energies that will govern important phenomena such as polymorphism and self-assembly, magnifying the importance of even small contributions. Such differences often depend on comparing molecules in very different environments. For example, the lattice energy of a molecular crystal is the balance between the energy of the solid lattice and that of a single isolated molecule. The vast difference in environments make collective effects that depend on an atom's or molecule's surroundings all the more important. Pairwise-additive models of vdW interaction will generally favour denser clusters and crystals, ignoring the contributions of specific arrangements of atoms and molecules in a molecular material. As such, pairwise-additive models can fail to model molecular materials both quantitatively and qualitatively. Quantitatively, ignoring collective effects leads to many pairwise methods overestimating cohesive energies of molecular crystals^19,41^ and host–guest systems,^21,42^ while also failing to capture the scaling of vdW interactions in different nano-materials, such as fullerenes.^3,43^ Qualitatively, many approaches fail to predict the correct polymorphic ordering of molecular crystals of even relatively small molecules.^18,20^ In the following section we will introduce a number of approaches to capturing many-body or collective effects in dispersion interactions. In Section 4 we will highlight the application of these methods to a number of the examples given above, showing how collective models of vdW interactions can overcome the limitations of pairwise methods.

## 3 Beyond pairwise-additive models for van der Waals interactions

Despite the widespread and often successful application of pairwise-additive models of van der Waals interactions, it has long been known that they represent only an approximation to the true many-body nature of dispersion interactions. For this reason, a number of approaches have been developed to capture many-body contributions to vdW interactions, ranging from simple three-body terms added to the pairwise expression to treatments that consider the collective nature of vdW correlations in a more fundamental manner.

### 3.1 Triple-dipole contributions

Given the origin of the pairwise approaches in second-order perturbation theory, the natural next step is to consider higher-order perturbative contributions. The Axilrod–Teller–Muto (ATM) three-body term considers triple-dipole energy contributions:^1,44,45^
6
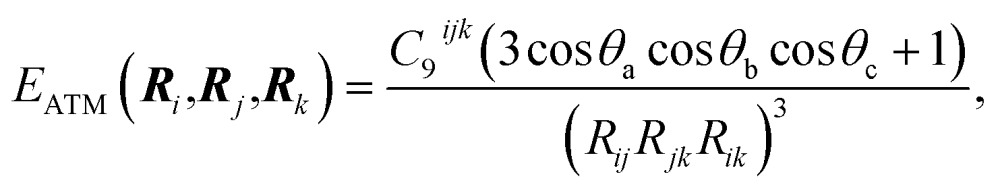
where the three angles *θ*_a_, *θ*_b_ and *θ*_c_ are the internal angles of the triangle formed by the three atoms *i*, *j* and *k*, and *C*_9_^*ijk*^ is the triple-dipole coefficient, which can also be obtained by integration of dipole polarisabilities over imaginary frequency,^1^ but are also estimated from *C*_6_ coefficients.^30^ Unlike the pairwise term, the ATM expression can give both positive and negative contributions depending on the triangle formed by the atoms, although the major contribution is a repulsive one for an equilateral triangle, which is a common occurrence in close-packed solids.^1,44^ The repulsion for an equilateral triangle arises because any interaction (*e.g.* formation of instantaneous and induced dipoles) between any two atoms of the triangle is frustrated by simultaneous interactions with the third, overall reducing the interaction compared to three distinct pairs of atoms.

The application of the ATM term has varied in different fields, as therefore has the understanding of its role and contribution. Its application to rare-gas solids yields a qualitatively correct picture of stability and elastic properties where pairwise methods fail.^46^ However, as evaluating eqn (6) for a collection of atoms necessitates a triple sum over the atoms, at least in former times its used widespread use was prohibitively expensive for force-field simulations.^7^ In the field of vdW-inclusive DFT such costs are negligible compared to the self-consistent cycle of the DFT calculation and a number of groups have investigated their use.^26,30,47^ As we shall see below, the performance and importance of the ATM expression has been mixed. Damping the ATM contribution and matching it with pairwise contributions or a density functional are likely to be part of the reason for this,^26^ but it is also the case that the ATM expression is just the first in a series of perturbative many-body contributions to vdW energy. Each of these terms can have alternating signs and analytical expressions of higher-order interactions become increasingly more cumbersome.^1,48^ A deeper understanding of collective vdW effects requires going back to the definition of polarisability of molecules with many atoms, and this is what we do in the next section.

### 3.2 Non-additive effects in the polarisability

To better understand how collective behaviour can affect vdW interactions we need to step back and consider the definition of polarisability of molecular materials. The polarisability of a molecule or material relates the extent to which its electronic structure is distorted by an external electric field. Specifically, the dipole polarisability, ***α*** relates an induced dipole moment to the applied electric field:

7 ***μ*** = ***αE***,

where ***μ*** is the dipole moment of the atom and ***E*** is the local field. The dipole polarisability is a 3 × 3 tensor by construction. Its non-local nature is critical to modelling polarisation and induction effects in molecular materials, which has been an active area of research and method development for many years.^1,4,6,49,50^ However, this aspect of polarisability and the importance of polarisation have largely been ignored in models of vdW interactions, despite the central role of polarisability in such interactions [*cf.*eqn (2)]. Accurately modelling vdW interactions requires considering polarisability and polarisation consistently.

Such a collective model of polarisability and polarisation can be achieved using classical electrodynamics, where the interaction between a collection of dipole polarisabilities can be modelled as:^1,6,51^
8
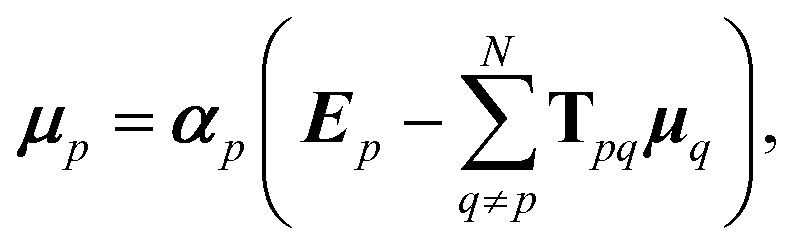
where ***μ***_*p*_ is the dipole moment of the *p* ^th^ atom, ***E***_*p*_ is the local field at *p*, and the term in brackets denotes the overall electric field due to coupling with the dipole of the *q*th atom, through the dipole–dipole coupling tensor **T**_*pq*_. Although this model is classical, the input polarisabilities and definition of the coupling tensor can be based on a quantum-mechanical description. The coupling tensor is normally defined as:

9 **T***ijpq* = ∇_*i*_∇_*j*_*V*(**r**_*pq*_),

where *i* and *j* represent the Cartesian components of the vector joining *p* and *q*, and *V* is the Coulomb potential.

It is possible to rearrange this expression and cast it in a matrix form so that an effective self-consistently screened (SCS) polarisability tensor can be defined that captures the effect of coupling between all the dipoles:^6,51^
10
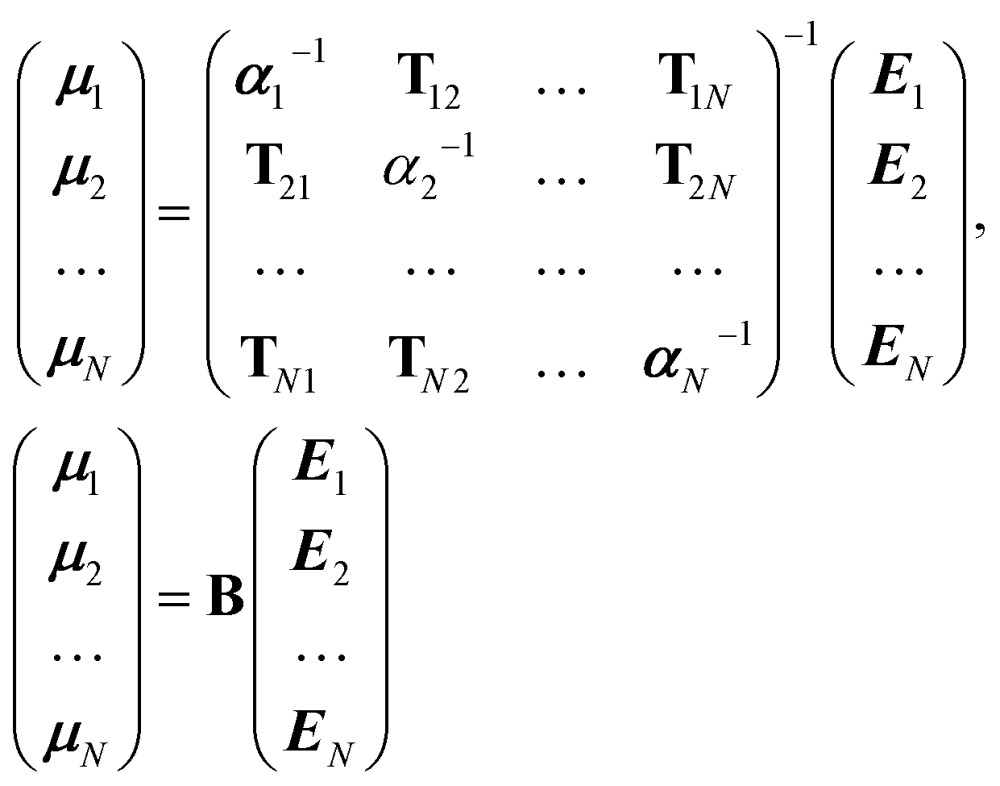



The effective polarisability matrix, **B**, is often referred to as the relay matrix.^6,51^ The effective “screened” polarisability of *e.g.* the molecule or crystal, is obtained by fully contracting **B**. Alternatively, the polarisability can be partitioned among the atoms, with one practical scheme being to row or column contract **B**:^23^
11
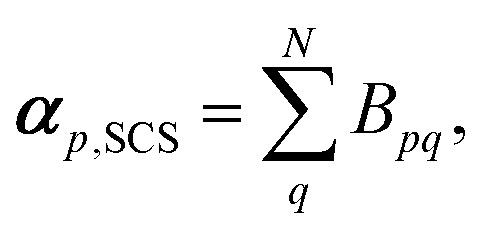
where *B*_*pq*_ is a 3 × 3 block of the 3*N* × 3*N* relay matrix. The result is an effective polarisability for each atom or fragment, which even if the input polarisabilities are isotropic is in general anisotropic due to the environment of each atom. Some care must be taken in the definition of the **T**_*pq*_, as the bare Coulomb potential (1/|**r**_*q*_ – **r**_*p*_|) can lead to non-physical interactions between atoms at short separations, where the distributed nature of the atomic dipoles must be properly accounted for.^1^ Damping of **T**_*pq*_ can yield reasonable results,^6^ but modelling each dipole as having an isotropic Gaussian distribution (corresponding to an isotopic quantum harmonic dipole oscillator) gives a simple but remarkably useful model.^23,24,49^ The above treatment can be performed using frequency-dependent polarisabilities, leading to frequency-dependent screened polarisabilities and hence screened *C*_6_ coefficients *via*eqn (2).^23^ Typically, this is done by first obtaining the average of the eigenvalues of the screened polarisability tensor for each atom. As the TS polarisabilities are functionals of the local electron density and already contain the important short-ranged effect of hybridisation,^22,36^ they are an ideal choice for the input polarisabilities in eqn (10), giving rise to the TS + SCS method for obtaining screened polarisabilities and *C*_6_ coefficients, which can be used in a pairwise energy expression.

**Fig. 1:**
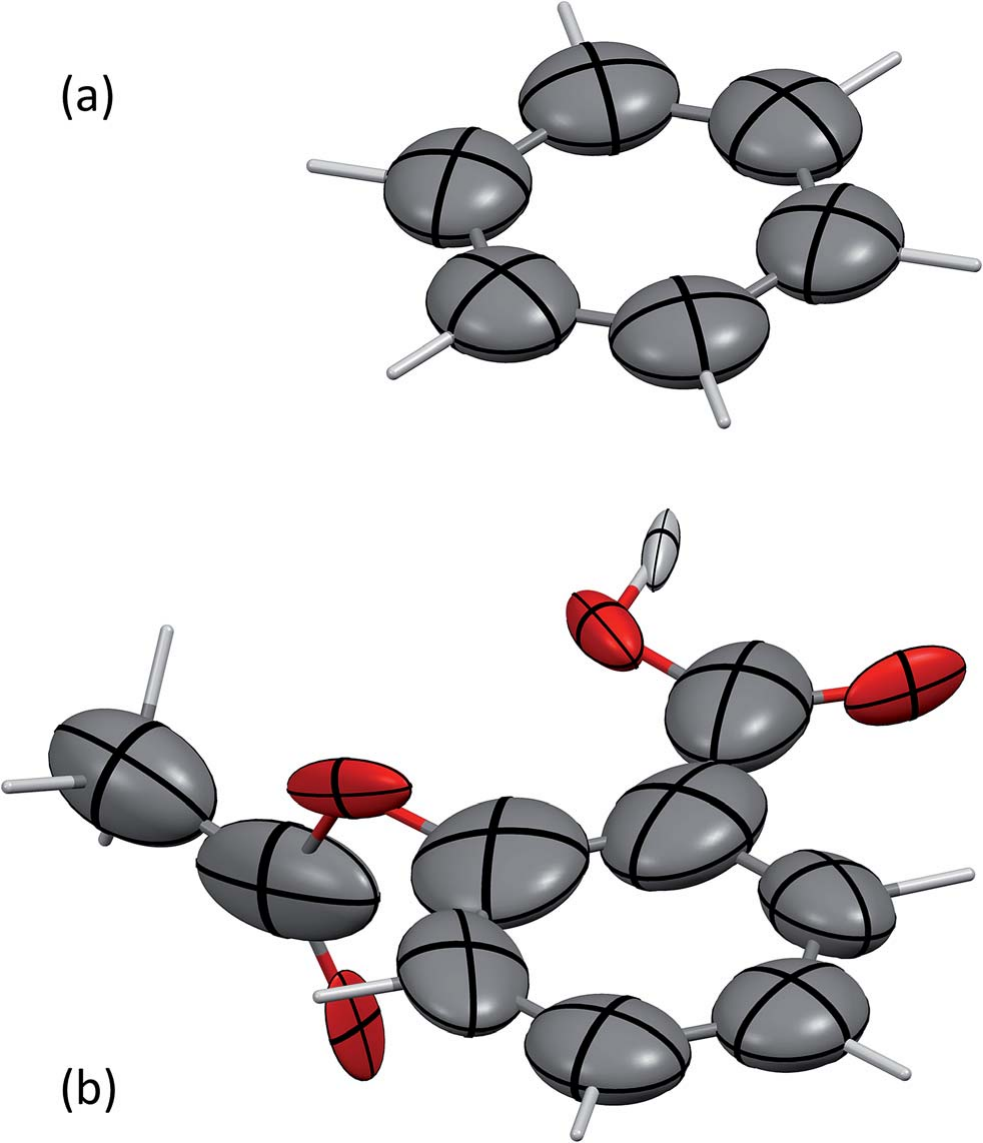
The anisotropic, screened polarisability tensors of (a) benzene and (b) aspirin. The tensors are represented as ellipsoids. Aliphatic H atoms are very strongly polarised towards their neighbouring C atom and are not shown for clarity. *Mercury* 3.4 has been used to produce these figures.^55^

The screened polarisabilities and *C*_6_ coefficients readily illustrate deviations from the isotropic model of polarisability and *C*_6_ often used in force fields and vdW-inclusive DFT methods. The polarisability tensor should be a positive semi-definite 3 × 3 matrix, which can be represented by an ellipsoid, in the same fashion as anisotropic displacement tensors are depicted in crystallography.^52^ Fig. 1 shows the screened polarisability tensors of benzene and aspirin as ellipsoids. In benzene, strong coupling in the plane of the molecule leads to an increase of in-plane polarisability and a corresponding reduction of out-of-plane polarisability. This is observed experimentally in the molecular polarisability with the out-of-plane polarisability being 55% of the in-plane value,^6^ which is correctly captured by TS + SCS polarisabilities but not by the TS ones.^25^

For aspirin the same in-plane polarisation within its benzene ring can be observed, although now the different groups surrounding the ring distort the polarisabilities to different extents. Aspirin is a useful illustration of two competing polarisation effects of electrodynamic screening. Within a molecule the effect of collective vdW interactions on *C*_6_ coefficients is to increase them, as atoms polarise strongly towards neighbours, with the average *C*_6_ coefficient of the C atoms in aspirin being 43.3 a.u. compared to 31.0 a.u. for TS. However, in the solid state, coupling with the surrounding molecules limits an atom's ability to polarise with its neighbours, resulting in net depolarisation. This leads to an average C-atom *C*_6_ of 39.0 a.u. in aspirin, a reduction of around 10% relative to the gas phase.^53^ Comparing these coefficients to values from condensed-phase force fields is difficult, as they typically involve balancing dispersion, repulsion and electrostatic contributions and frequently have very different values. For example, an aromatic C···C interaction in the W99 force field has a dispersion coefficient of around 30 a.u.,^33^ whereas in the UNI force field all C···C dispersion interactions have a coefficient of 42 a.u.^54^

**Fig. 2:**
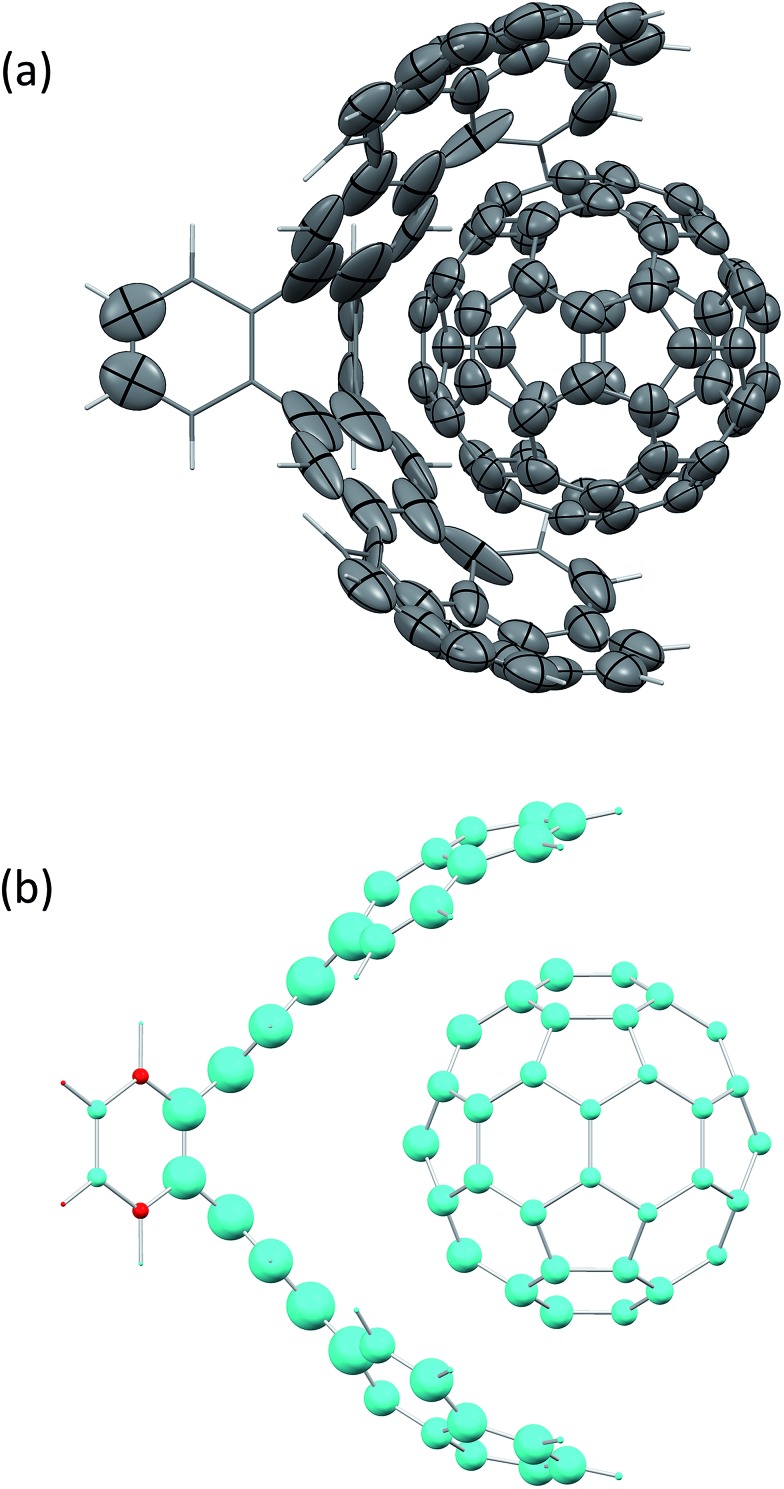
(a) The anisotropic, screened polarisability tensors of a “buckyball catcher” host–guest system and (b) a plot of the difference between *C*_6_ coefficients for the whole complex and the separate parts (in the same geometry).^56^ Some atoms polarise so strongly that they cannot be represented by an ellipsoid in (a). The size of the spheres in (b) indicates the magnitude of the difference, with blue meaning a decrease in *C*_6_ upon forming the complex and red denoting an increase in the complex.

Fig. 2(a) shows the screened polarisability tensors of a “buckyball catcher” host–guest system (C_60_@C_60_H_28_).^56^ The host molecule exhibits dramatic departures from a model of spherical and near identical polarisabilities for each C atom. In some cases the polarisation is so strong and deviates so much from the isotropic model that the polarisability tensor is not positive semi-definite, although it is still possible to calculate *C*_6_ coefficients for these atoms. Fig. 2(b) shows the change in TS + SCS *C*_6_ coefficients when the complex is formed. The blue spheres denote atoms that depolarise when the complex is formed, leading to a smaller *C*_6_ coefficient. This depolarisation is strongest at the interface between the two molecules and there is a clear asymmetry to the C_60_ molecule. Using the TS + SCS *C*_6_ coefficients in a pairwise expression for the dispersion energy for this complex this yields a reduction of the binding energy of the order of 30 kJ mol^–1^ compared to TS,^56^ illustrating the importance of capturing these depolarisation effects in cohesion. The TS + SCS approach to pairwise vdW energies has been applied to a number of systems by Bučko and co-workers,^57^ and has shown that electrodynamic screening is important in a variety of situations.

### 3.3 van der Waals correlations from the ACFD theorem

The self-consistently screened polarisabilities and *C*_6_ coefficients discussed above capture important collective effects in vdW interactions, as does the ATM expression for triple-dipole contributions. However, the accurate treatment of vdW interactions requires methods than can capture both screening and many-body effects in a systematic manner.^22^

Within the framework of the adiabatic connection fluctuation-dissipation (ACFD) theorem there is an exact expression for the correlation energy in terms of density–density response functions *χ*(**r**,**r′**,*iω*), which describes the density response at **r′** as a result of a perturbation at **r**.^15^ The correlation energy of a system can be written in terms of the response functions of a corresponding mean-field system (*χ*_0_), and the response functions of a corresponding interacting system, where the Coulomb interaction *v* is scaled by a parameter *λ*:^58^
12
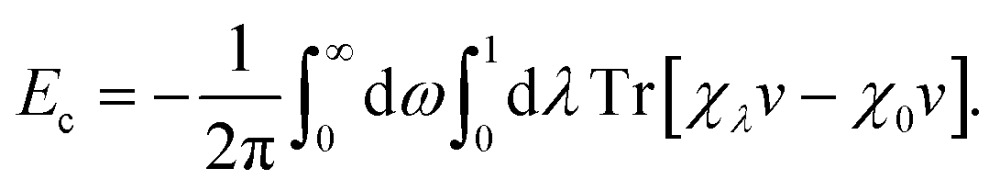



The bare response function (*χ*_0_) can be determined from the eigenfunctions and eigenvalues of an electronic-structure calculation,^59^ but some approximation is required for the interacting response functions. Within the popular random-phase approximation (RPA) the interacting response functions are approximated in terms of the non-interacting ones as follows:^15,58^
13
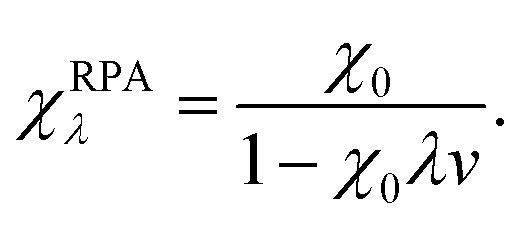



RPA is commonly employed with DFT or HF orbitals and contains the ring diagrams of coupled-cluster theory with double excitations. RPA can have remarkable performance for many properties in a variety of situations,^15,60^ particularly for long-range correlation and dispersion, but has a substantial computational cost, scaling normally as *N* ^4^ or higher,^61^ which has limited its widespread use. However, a recent *N* ^3^ algorithm may increase its use.^62^

### 3.4 The many-body dispersion (MBD) method

The ACFD theorem gives a route to the correlation energy of a system in terms of response functions. While it is natural to consider the full response of a system based on a full and accurate representation of its electronic structure, model response functions could also be used to capture non-local correlation energy.

It is possible therefore to obtain an accurate but efficient description of the long-range many-body van der Waals interactions by combining the ACFD expression for the correlation energy with response functions that model only the dipole response of an atom. This is the approach adopted by the many-body dispersion (MBD) method,^23^ where the correlation or dispersion energy is given by:^24^
14
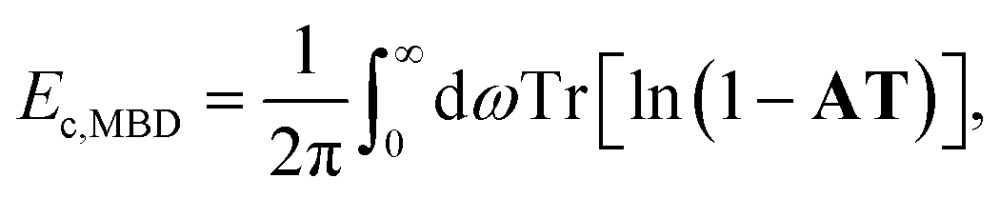
with **T** being an appropriate dipole–dipole coupling tensor and **A** is a diagonal 3*N* × 3*N* matrix, with each block derived from an appropriate isotropic distributed polarisability: **A**_*lm*_ = –*δ*_*lm*_ *α*_*l*_(*iω*). In this expression the dipole response of each atom is modelled using a single localised, isotropic quantum harmonic dipole oscillator. The RPA treatment of this model considers all orders of dipole coupling at long range, including triple and higher dipole energies and collective screening simultaneously and systematically.^23,24,63^ Due to the localised description of the response or polarisability, which is centred on atoms, the MBD method is ideally suited to materials or systems with a finite band gap. The ACFD theorem expression can be recast as a straightforward Hamiltonian expression,^63^ which can then be directly diagonalised using simple and efficient matrix algebra. The contribution of the MBD energy to atomic forces is also straightforward to derive and implement.^24^

The MBD method has to-date largely been coupled with density functional theory,^23,64,65^ where the underlying functional accounts for short-range correlations. Therefore, some form of range separation or damping of the MBD correlation energy is required to avoid double counting, so that the MBD energy is curtailed at short distances. This is reflected in the input polarisabilities and the definition of the dipole–dipole coupling tensor. The polarisabilities are typically obtained in a two-step process where short-range hybridisation effects are considered by using TS polarisabilities and screening contributions are evaluated using the self-consistent screening equations [eqn (8)]. More rigorous range separation can be achieved by considering only short-range screening contributions, as in the MBD@rsSCS method,^24^ and in general this improves the performance of the method. A more in-depth discussion of the theory can be found in a number of recent publications.^24,25^

MBD has been combined with DFT largely due to the balance of accuracy and computational cost that this combination achieves, as we shall discuss below. However, MBD could be used to provide a model of long-range correlation or dispersion in other methods including empirical approaches such as force fields. Bereau and von Lilienfeld have recently introduced a scheme using MBD with polarisabilities derived from Voronoi tessellation.^66^

## 4 Collective vdW interactions in dispersion coefficients, energies and response properties

Pairwise and collective models of vdW interactions have been applied in a wide variety of situations. Taking examples from small molecules, supramolecular systems and molecular crystals, in this section we highlight the importance of collective vdW methods in accurately modelling molecular materials.

### 4.1 Collective effects in polarisabilities and dispersion coefficients

We begin by briefly discussing the role of collective effects in polarisabilities and dispersion coefficients of atoms and molecules in different environments. For small molecules, pairwise-additive methods can capture *C*_6_ coefficients quite well, with the TS method has an accuracy of 5.5% for a set of 1225 small-molecule *C*_6_ values,^67^ and other pairwise methods and vdW density functionals have values in the range of 8–30% using the same or comparable test sets.^30,68,69^ However, the underlying polarisabilities of small molecules can still deviate substantially from the isotropic model employed by pairwise-additive approaches, as seen above for benzene and aspirin in Section 3.2. For 18 small molecules the TS method has a mean absolute relative error of 76.3% for the fractional anisotropy of the molecular polarisability with respect to experiment.^25^ Including self-consistent screening, as in the TS + SCS method, captures a substantial portion of this anisotropy, reducing the error to 33.5%.

In molecular crystals the role of collective effects in molecules is more complex. As noted for aspirin above (Section 3.2), short-range polarisation within molecules increases *C*_6_ coefficients while long-range depolarisation due to surrounding molecules decreases them. The acene crystals are an excellent example of this, with average C-atom *C*_6_ coefficient increasing from naphthalene to pentacene for the isolated molecule but decreasing in the solid as the molecule grows in size.^70^ Capturing these effects is important for predicting the dielectric constant and optical properties of the acene crystals. The TS method overestimates dielectric constants substantially, while TS + SCS is much closer to experiment and benchmark calculations.^70,71^ Screened *C*_6_ coefficients of a set of semiconductors obtained from time-dependent DFT (TDDFT) have also been shown to differ significantly from pairwise values, with a pairwise vdW term based on screened *C*_6_ coefficients noticeably improving the cohesive properties of the semiconductors.^72^ The importance of defects and vacancies has also been studied in semiconductors (using TS + SCS), showing that *C*_6_ coefficients are significantly altered by their presence, affecting stability and defect migration barriers.^73^

Collective effects in nanostructures lead not only to changes in *C*_6_ coefficients but their fundamental scaling with system size. For a pair of fullerenes pairwise-additive models predict a *C*_6_ coefficient between the two fullerenes that scales as *n* ^2^, where *n* is the number of C atoms in the fullerene. A number of recent studies have shown larger power laws. TS + SCS gives scaling of *n* ^2.35^,^3^ while modelling the polarisability using spherical shells of valence electron density yields a power law in the asymptotic limit of *n* ^2.75^.^43^ TDDFT calculations of smaller fullerenes shows a power law of *n* ^2.2^.^74^ *C*_6_ coefficients in other nanomaterials, such as multilayer graphene and carbon nanotubes also feature large and non-linear changes with system size that are not captured by simple pairwise methods.^3^ This behaviour stems from the high symmetry and reduced dimensionality of these systems (*e.g.* “0-D” fullerenes; “2-D” graphene), which lead to very strong polarisation effects. For layers of graphene sheets, such effects lead to stronger interactions within sheets, which then reduces binding between sheets. TS overestimates the binding between sheets substantially [87 meV per C atom *versus* an experimental estimate of 52(2) meV per C atom]. In contrast, PBE0 + MBD and RPA calculations agree much better with experiment, with values of 50 meV per C atom and 48 meV per C atom, respectively.^24,75^

While the role of non-additive behaviour in *C*_6_ coefficients is minor in small molecules, it is clear that as we move to solids and nanomaterials these effects become much more pronounced, changing not only absolute values but also altering fundamental scaling and behaviour. In the following subsections we will discuss how this manifests itself in cohesive energies and properties.

### 4.2 Collective vdW interactions in molecular assemblies

Isolated molecular assemblies, ranging from small molecular dimers to supramolecular host–guest complexes, are amongst some of the most widely used benchmarks used to evaluate and understand vdW interactions and their contribution to cohesion. For small molecular dimers there are a number of databases of coupled-cluster singles, doubles and perturbative triples [CCSD(T)] binding energies, representing a “gold-standard” benchmark with which to compare different approaches. The S22 and S66 databases of Hobza and co-workers contain 22 and 66 dimers,^10,11^ respectively, comprised of hydrogen-bonded interactions, vdW interactions and mixed bonding. These databases have been used to assess pairwise *C*_6_/*R* ^6^ methods,^53,76^ many-body dispersion (MBD)^24,53^ and also vdW density functionals.^77^ In general, many pairwise methods perform well for these databases, yielding mean absolute errors (MAEs) of 1–2 kJ mol^–1^, better than the chemical-accuracy target of 4.2 kJ mol^–1^. In comparison, semi-local functionals without pairwise vdW contributions can yield errors greater than 8 kJ mol^–1^,^76^ highlighting the importance of these interactions, even for simple systems. Considering collective vdW contributions can further improve upon the performance of pairwise methods,^23,53^ but in general their role in these small systems is not dominant.

In larger supramolecular systems, collective effects are clearly more important. The S12L dataset of Grimme^12^ comprises 12 supramolecular host–guest complexes. Risthaus and Grimme found that including at least three-body vdW contributions (from the ATM expression) was necessary for these systems, with contributions of the order of 10 kJ mol^–1^. The “buckyball catcher” in Fig. 2 has a contribution of 13.4 kJ mol^–1^ from the ATM expression.^21^ Three-body contributions generally improved pairwise methods, although not consistently, with D3 coupled to the PBE functional^78^ having a mean absolute error for binding energies of 8.8 kJ mol^–1^ but PBE + D3 + *E*_ATM_ having an MAE of 10 kJ mol^–1^. The pairwise Tkatchenko–Scheffler vdW method yields an MAE of the order of 30 kJ mol^–1^,^24^ despite easily reaching chemical accuracy for small molecules. Including collective vdW contributions using the MBD method reduces this error dramatically to around 6.7 kJ mol^–1^, approaching close to the statistical error of benchmark diffusion Monte Carlo binding energies for a subset of the S12L.^42^

Decomposition of the many-body contributions in the S12L shows a significant contribution from three-body interactions, in line with the contribution seen with the ATM expression,^21^ but the series only starts to converge with six-body contributions.^42^ These are not short-ranged interactions of the same nature as the ATM-type three-body term, but rather the effect on the dispersion energy of coupling and polarisation of groups of six atoms *via* more long-ranged 1/*R* ^3^ terms, as in eqn (10). In the C_60_@C_60_H_28_ system, even atoms in the C_60_ molecule far from the host molecule are affected by screening, as can be seen from the change in *C*_6_ coefficients in Fig. 2(b). As the many-body expansion typically alternates between positive and negative energy contributions, truncating at a lower order could lead to spurious over or under binding. Similar high-order contributions in DNA helices, where pairwise methods can omit more than 20% of the full many-body vdW energy.^79^ Many-body contributions have also been found to be important for describing the conformational preference of the Ac-Phe-Ala_5_-LysH^+^ peptide chain.^80^ It is clear from these studies that modelling the binding of supramolecular systems requires some model of collective and many-body vdW interactions.

### 4.3 Collective van der Waals effects in molecular solids

Molecular solids represent a diverse and challenging class of systems for applying different descriptions of vdW interactions to different properties, ranging from lattice energy to elastic properties and phonons.

#### 4.3.1 Benzene crystal

Benzene is naturally one of the most widely studied molecular crystals. Recently, several groups have studied the lattice energy of benzene and have found it important to include many-body contributions.^47,81–84^ One approach is to expand the lattice energy as a mean-field contribution (*e.g.* a HF energy) plus a summation of contributions from dimers, trimers *etc.* within the lattice, calculated at a high level of theory. As MP2 cannot capture three-body dispersion contributions, Kennedy *et al.* have been able to estimated the three-body dispersion contribution by comparing MP2 energies for over 350 trimers from the crystal with CCSD(T) energies and using the ATM expression for further more-distant trimers.^81^ The contribution is of the order of +3.2 kJ mol^–1^ (*i.e.* reducing the magnitude of the lattice energy), around 6% of the lattice energy, but smaller than previous estimates based solely on the ATM expression with different *C*_9_ coefficients, which range from 3.8–7.0 kJ mol^–1^.^47,81,82^

Yang *et al.* have used a similar expansion (including dimers, trimers, tetramers and additional correlation contributions) to estimate the benzene lattice energy as –55.9(9) kJ mol^–1^, which compares very well with an experimental estimate of –55.3(22) kJ mol^–1^ obtained from sublimation enthalpies and theoretical zero-point contributions.^84^ This value involved not only a significant three-body contribution to the lattice energy but a four-body one as well. It is worth noting that “body” is a complete benzene molecule in these approaches, and thus these many-body contributions are not directly comparable to the three-atom contributions of the ATM expression.

The MBD method has also been applied to benzene, where the difference between the TS pairwise lattice energy and the MBD lattice energy is around +11 kJ mol^–1^ when both are coupled to either the PBE or PBE0 (ref. 85) density functionals, reducing the lattice energy to –55.0 kJ mol^–1^ for PBE + MBD or –51.2 kJ mol^–1^ with PBE0 + MBD.^41^ The pairwise TS method, which is accurate to 1.5 kJ mol^–1^ for small-molecule binding energies and 5.5% for *C*_6_ coefficients,^36,53^ significantly overestimates the lattice energy in the absence of collective screening and many-body energy contributions. The role of these effects is even more pronounced when considering larger oligoacenes such as naphthalene and anthracene.^41,70^

#### 4.3.2 X23 dataset

Recent efforts to develop benchmark dataset such as the C21 (ref. 19) and the X23 (ref. 41 and 53) have permitted a broader assessment of absolute lattice energies with various methods. The X23 is based on the C21 database and contains a mixture of hydrogen-bonded solids, vdW-bound solids and solids with a mixture of the two interactions. Experimental lattice energies were determined from experimental sublimation enthalpies,^86^ theoretical vibrational contributions (including vdW contributions) and, where available, experimental heat capacities, with an expected accuracy just better than chemical accuracy (4.2 kJ mol^–1^), which is largely limited by experimental uncertainties in sublimation enthalpies.^41^

**Fig. 3:**
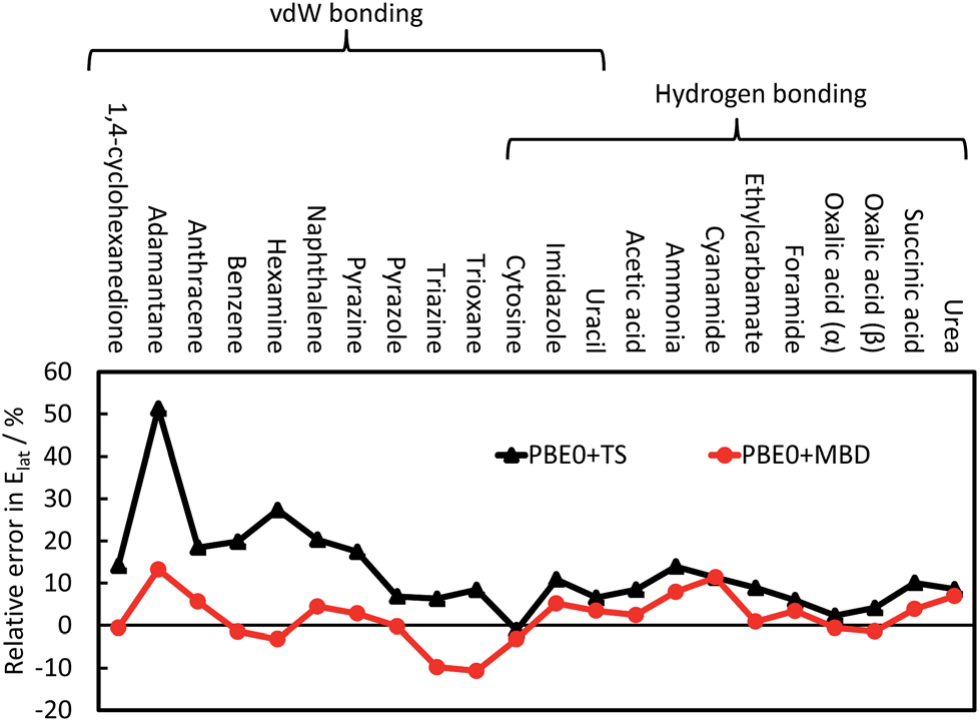
The relative error in the calculate lattice energies for 22 vdW-bound and hydrogen-bonded systems. A positive value indicates overestimation of the experimental lattice energies; see ref. 41 for more details and a discussion on CO_2_.

The TS pairwise method has a mean absolute error (MAE) for the X23 of 10.0 kJ mol^–1^ when coupled with the PBE0 functional, systematically overestimating lattice energies with an accuracy nearly an order of magnitude worse than that found for small dimers in the S22. Including many-body contributions with MBD yields an MAE of 3.9 kJ mol^–1^, within the chemical-accuracy limit. The reduction in lattice energies is particularly pronounced for vdW-bound systems, as can be seen in Fig. 3, but is still important for hydrogen-bonded systems. The reduction in lattice energies arises from long-range depolarisation of vdW interactions, as seen for *C*_6_ coefficients (Section 3.2) and repulsive many-body contributions, as would be *partly* obtained with the ATM expression (Section 3.1).

A number of other pairwise approaches have been tested with the C21 and X23 databases, including XDM, D2 and D3.^19,41,87^ These methods have better MAEs than the TS pairwise approach, with D3 coupled with the TPSS meta-GGA functional^88^ being capable of reaching chemical accuracy as well. However, these methods typically have a more flexible and empirical approach to fitting and determining the *C*_6_ coefficients and damping of the short-range vdW interactions.^19,29,76^ For example, in the D2 approach there is a global and empirical scaling of *C*_6_ coefficients as part of the fitting process for the density functional employed. D3 and XDM both use the Becke–Johnson damping function,^89^ which features an additional fitting parameter compared to TS and does not damp the vdW contribution to zero as *R* → 0. The TS and MBD approaches are, in part, fitted to the S22 and S66 sets of small dimers to avoid including effects from larger molecules. D3 employs a wider set of benchmark data,^30^ while in XDM these parameters are often re-fitted for a specific basis set,^90^ potentially factoring in other contributions into the parameters.

Many of the empirical adjustments used in pairwise approaches would improve the performance of the TS method for molecular crystals, which we have noted before in the case of a global scaling of the TS *C*_6_ coefficients for solids.^41^ However, such adjustments limit the transferability of methods, creating specific approaches for different materials. As noted above, Risthaus and Grimme advocate the use of the ATM expression in host–guest systems. For molecular solids, using the ATM expression has proven difficult though. It requires an alternative form of the XDM method's damping function, worsening the underlying pairwise MAE and not yielding any additional improvement.^26^ For D3, the three-body contributions worsens the MAE for the TPSS functional by about 1.3 kJ mol^–1^ but improves the HSE06 functional^91^ result by 0.8 kJ mol^–1^.^87^ These inconsistent results with D3 and XDM methods stress the need for methods that model vdW interactions seamlessly and systematically across a range of different sizes and forms of molecular materials. By including collective vdW effects, MBD is able to systematically improve upon the performance of the TS method, providing an accurate method with minimal empiricism.

The non-local vdW-DF and vdW-DF2 functionals^39,92^ have also been applied to the C21 set and show better performance than TS but worse than MBD or XDM, with MAEs of 6.1 kJ mol^–1^ and 6.4 kJ mol^–1^, respectively.^19,41^ Such functionals model the long-range correlation by performing a double integral over the whole electron density, summing contributions to the vdW using an interaction kernel that has a 1/*R* ^6^ dependence at long range. Much of the work in improving these functionals has focussed not on the non-local part but on the semi-local functional used alongside it, as the performance of these methods is sensitive to the coupling between the non-local correlation and the semi-local functional used.^14,92^ This can be exploited to tune these functionals to certain types of systems.^93^ However, this leads to a broad spectrum of vdW-DF-based methods with their performance wildly varying across a range of molecular materials.

#### 4.3.3 Molecular-crystal polymorphism

The importance of many-body contributions extends from lattice energies to relative lattice energies and the challenge of polymorphism, where energy differences in the range of 0.1–1.0 kJ mol^–1^ need to be resolved.^8,94^ On such scales even small many-body or collective vdW contributions can yield different predictions for the most stable polymorph or the low-energy portion of the polymorphic landscape.

**Fig. 4:**
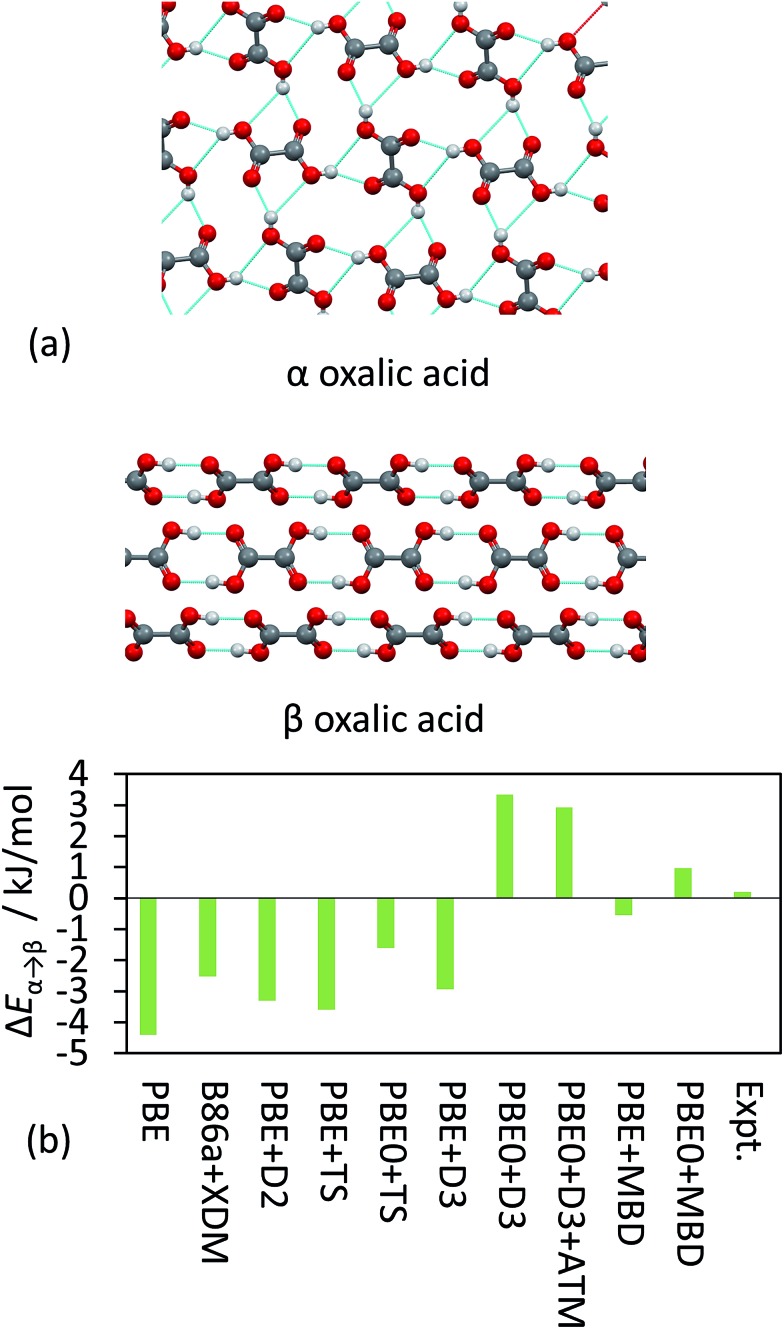
(a) The hydrogen-bonding interaction in α and β oxalic acid. (b) The relative energies between the polymorphs from experiment and as obtained with a number of different DFT methods.

TS and MBD have been applied to a number of polymorphic systems, demonstrating the importance of collective effects in these systems. Oxalic acid has two known forms, α and β [Fig. 4(a)]. Experimentally, the α form is found to be slightly more stable by around 0.2 kJ mol^–1^ in terms of lattice energy.^19,41,95^ A number of pairwise vdW methods used in conjunction with DFT (including TS, XDM and D2)^19^ systematically overestimate the stability of the β form by up to 4 kJ mol^–1^ [Fig. 4(b)]. Accounting for collective effects using MBD and employing the non-empirical hybrid functional PBE0 yields the correct qualitative ordering. The D3 approach also yields the correct ordering in conjunction with PBE0 but has a much larger energy difference than observed experimentally,^87^ while vdW-DF2 is also able to reproduce the correct ordering (note however that this result will depend on the employed semi-local approximation to the exchange–correlation functional).^19^ It is worth noting here that the best result in Fig. 4(b) in terms of absolute difference (PBE + MBD) represents the wrong ordering of the polymorphs. As methods often aim to minimise MAEs it is important that trends are still captured correctly. TPSS + D3 yields a smaller MAE for the X23 than PBE0 + D3 but incorrectly predicts β oxalic acid to be more stable.^87^

Glycine is a challenging polymorphic system where computational methods often have difficulties. An experimental ordering has been established for three known polymorphs of glycine, with the γ one being be the most stable, followed by α and then β. The TS pairwise method systematically overestimates the stability of the α and β forms, while again including collective effects the correct experimental ordering is obtained with PBE0 + MBD.^20^ The potential-energy surface and geometry of glycine is also markedly affected by many-body contributions.

Finally, oxalyl dihydrazide is another system where many-body contributions have been explored, although only in the three-body ATM form. Two separate studies have shown the ATM expression affecting relative energies by more than 1 kJ mol^–1^,^18^ which in one case leads to a different ordering of the polymorphs.^18,96^ The difference may arise in the damping of the three-body interaction or the manner in which the *C*_9_ coefficients are determined.

The importance of collective vdW interactions in these systems is interesting, as they would normally be thought of as small hydrogen-bonded systems, where vdW interactions are very much a secondary concern. In larger systems the importance of vdW interactions can grow and with it the role of collective vdW interactions. In Section 3.2 the significant effect of screening on the *C*_6_ coefficients of aspirin was highlighted. Overall, the MBD lattice energy for form-I aspirin is reduced by nearly 12 kJ mol^–1^ relative to the TS pairwise value.^53^ Wen and Beran have estimated the 0 K lattice energy difference between form-I and form-II aspirin to be of the order of ±0.1 kJ mol^–1^ using MP2-based methods.^97^ MBD agrees with this, yielding an essentially degenerate lattice energy difference for the two forms. TS gives an energy difference of –0.2 kJ mol^–1^ in favour of form II (Δ*E* = *E*_I_ – *E*_II_). Despite the lattice energy change of nearly 12 kJ mol^–1^, the relative energies are very similar with the pairwise and many-body treatment. However, as we shall see below, going beyond the 0 K lattice energy reveals substantial differences between the two vdW treatments.

#### 4.3.4 Beyond 0 K lattice energies

Many studies of polymorphism focus on lattice potential energies, devoid of any zero-point or vibrational contributions. However, the finite-temperature stability of a given structure is dependent on lattice free-energy differences at a given temperature. In the harmonic limit these can be readily determined from phonon or solid-state vibrational spectra of the different polymorphs.^98,99^

As noted above, the two known forms of aspirin^100^ are degenerate in terms of lattice energy when calculated with a variety of different methods. However, while intergrowth of the two forms occurs,^101^ form I appears to be the more thermodynamically stable and abundant form.^102^ The free-energy difference between the two forms at 298 K is relatively small (–0.7 kJ mol^–1^, favouring form II) when calculated with the TS pairwise method. However, when the MBD method is used there is significant stabilisation of form I by 2.6 kJ mol^–1^,^99^ rationalising experimental observations. This arises due to softening of low-frequency vibrational modes in form I, making it entropically stabilised. The different arrangements of methyl groups and hydrogen bonds in the lattice may explain the ability of many-body interactions to influence the vibrations differently in the forms. Given the fact that the two forms are quite similar structurally (differing in weak C–H···O hydrogen bonds),^100^ such effects are likely to be found in other polymorphic systems where more significant differences can occur.

Dispersion interactions also play an important role in the elastic or mechanical properties of molecular materials, which are important for their processing and practical application, *e.g.* tabletting of pharmaceuticals.^103^ Recently, the importance of pairwise vdW interactions has been highlighted for peptide nanostructures.^104^ Aspirin also highlights the role of collective vdW interactions in elastic behaviour. With the TS pairwise method the bulk modulus of form-I aspirin is 12.40 GPa,^99^ compared to a value of 7.77 GPa at 300 K.^105^ Collective vdW contributions reduce the calculated bulk modulus by over 20% to 9.58 GPa, with a similar reduction for form II. Much of the remaining difference is likely to arise from vibrational contributions.^106^ The shear modulus is also reduced by over 20% by many-body contributions in both forms. The reduction in the strength of vdW interactions that arises to collective effects is more apparent in derived properties such as the elastic constants. This phenomenon has also been seen using the TS + SCS method, which has been used to determine the bulk modulus of rare-gas molecular crystals by Bučko *et al.* ^57^ For rare-gas crystals the screened pairwise method reduces bulk moduli by around 6–7%. For benzene, PBE with D2, TS, and TS + SCS all yield a bulk modulus of 10 GPa,^57,107,108^ overestimating a 0 K experimental estimate of 8.0 GPa by 25%.^109^ In contrast, RPA based on PBE orbitals (*E*_xx_/RPA@PBE) yields a value of 7.5 GPa,^110^ which is much closer to experiment.

While the focus of research in vdW interactions has largely been on cohesive energies and structure, much of the importance of molecular materials derive from their properties. The study of elastic properties and vibrations already suggests that collective vdW effects play an important role in governing the behaviour of molecular materials beyond their fundamental stability.

## 5 Future directions and challenges

In recent years our understanding of the importance and role of van der Waals interactions, especially those of a collective nature, has substantially increased. Here, we highlight some of the different aspects and areas of vdW interactions that still pose significant challenges, while simultaneously offering significant opportunities for new insights.

Considerable effort has been put into creating a number of benchmark datasets for assessing different approaches to non-covalent interactions,^10–12,19,41^ which have spurred development of new methods and our understanding of collective vdW interactions. These have largely focused on energetics and to a lesser extent geometry. For solids, obtaining suitable benchmark energies is challenging in the absence of a tractable high-level computational method. Benchmark data for derived properties, such as phonons and elastic properties, are even harder to obtain from theory alone and experimental data requires accounting for thermal and zero-point contributions. However, further understanding of the importance of vdW interactions hinges on new benchmark data to push and challenge computational methods. Gaining acceptance for new general-purpose benchmark datasets, as the S22 and S66 already are for small-molecule dimers, is also crucial.

The role of many-body and collective vdW interactions in liquids and solutions, which play a critical role in so much of chemistry, has not been explored to the same extent as for solids or isolated systems. Probing such systems requires sampling of their dynamic behaviour, which is computationally demanding from first principles. However, as the recent work shows, it is possible to decouple models of collective vdW interactions from DFT, allowing their use in empirical potentials.^66^ The Drude oscillator model has been used to model induction and vdW interactions in rare-gas liquids^27^ and water.^111^ For general use, correlation between models of vdW interactions and *e.g.* repulsion terms in force fields may make initial parameterisation and applications challenging. However, new force fields may lead to new insights into collective effects on scales not encountered in “perfect” molecular crystals or isolated supramolecular systems. For example, recent experimental work has shown that the role of dispersion in molecular recognition of apolar alkyl chains in solution appears to be much smaller than anticipated from pairwise models of vdW interactions,^112^ which might well stem from screening of vdW interactions.

Expanding the application of vdW-enabled methods will require extending and developing these collective vdW methods further. The MBD method at present considers only dipolar responses to the vdW energy, ignoring higher multipole contributions. While these contributions are short ranged compared to the dipole term, which appears to be dominant in many cases,^41,113,114^ higher multipole contributions may well be more important for molecular materials at high pressures, where atoms approach closer together. The model response functions used in MBD could be extended to capture such higher-order responses.^24,25^ Currently, the use of MBD requires a single empirically fitted range-separation parameter to couple it with a chosen density functional, just as pairwise methods require one or more parameters in their damping functions. Defining the range separation in terms of the functional itself or developing new functionals implicitly alongside MBD would remove this empiricism and may enhance its transferability. Another area of on-going development is the application of MBD to metallic systems. Due to its localised and atom-centred nature, MBD is ideally suited to systems with a band gap. Delocalised metallic states can lead to strong screening effects within the metal but also between surfaces and adsorbing molecules, as captured by the vdW^surf^ method.^115^ MBD could be extended to capture these effects by modelling delocalised states alongside localised atom-centred contributions, with Wannier functions being one possible route to achieve this.^64^

The importance of developing these methods and concepts further lies in the need to model, and ultimately design, hybrid materials that may contain different manifestations of collective vdW interactions. Such manifestations will need to be captured in a systematic and seamless manner to achieve this: an ongoing challenge for the vdW research community.

## 6 Conclusions

Dispersion or vdW interactions are critical for the stability of numerous molecular materials. However, many studies consider such interactions only in a pairwise-additive form (mainly consisting of *C*_6_/*R* ^6^ terms), neglecting collective effects that originate from the quantum-mechanical many-body electronic interactions they result from.

Considering these collective effects in the dipole limit can yield screened polarisabilities and *C*_6_ coefficients for atoms that can dramatically depend on the local and non-local environment of an atom or molecule, varying significantly from “one size fits all” *C*_6_ coefficients used in many pairwise approaches. Such environment-aware interactions could play an important role in molecular recognition and assembly. Beyond capturing such effects in the coefficients used in the pairwise expression, there are additional many-body energy contributions that need to be captured. The many-body dispersion method,^23^ captures both effects in an efficient RPA-like treatment of dispersion and non-local correlation, and has been used here to illustrate their role.

In small-molecule dimers collective effects are not essential for achieving accurate binding energies. However, in supramolecular systems even the simplest triple-dipole contribution (from the ATM expression) can yield corrections to binding energies of the order of 10 kJ mol^–1^.^21^ However, analysing the many-body energy suggests that contributions as high as sixth order are needed for converged interaction energies, as the collective effect of atoms on polarisabilities is much more long ranged than the three-body ATM energy contribution.

Many-body effects are also important in molecular crystals. Recent work by a number of groups has highlighted the need for capturing three- and even four-*molecule* interactions to accurately model the stability of the comparatively “simple” and symmetric benzene crystal.^81,84^ The MBD method corrects systematic overestimation of lattice energies found in the TS pairwise method and yields the correct polymorphic ordering for a number of challenging polymorphic systems. Adding three-body contributions to other approaches has yielded mixed results though, highlighting the need for methods that treat vdW interactions in a systematic and seamless manner.

More recent explorations of vdW interactions in response properties highlights the role of collective vdW interactions not just in stability but in the properties and function of molecular materials. More benchmark data are needed to fully explore these aspects of vdW interactions and there is also a need to move beyond static lattices and isolated systems to consider liquids, solutions, and disordered and dynamic systems.
